# Glycometabolic rearrangements--aerobic glycolysis in pancreatic cancer: causes, characteristics and clinical applications

**DOI:** 10.1186/s13046-020-01765-x

**Published:** 2020-11-30

**Authors:** Lidong Cao, Jiacheng Wu, Xianzhi Qu, Jiyao Sheng, Mengying Cui, Shui Liu, Xu Huang, Yien Xiang, Bingjin Li, Xuewen Zhang, Ranji Cui

**Affiliations:** 1 Department of Hepatobiliary and Pancreatic Surgery, Second Hospital of Jilin University, Changchun, 130041 China; 2 Jilin Engineering Laboratory for Translational Medicine of Hepatobiliary and Pancreatic Diseases, Changchun, 130041 China; 3 Department of Hepatobiliary and Pancreatic Surgery, the First Bethune Hospital of Jilin University, Changchun, 130021 China; 4 Jilin Provincial Key Laboratory on Molecular and Chemical Genetics, The Second Hospital of Jilin University, Changchun, China

**Keywords:** Malignant tumors, Pancreatic, Glycometabolic, Glycolysis, Exocrine

## Abstract

Pancreatic cancer is one of the most malignant tumors worldwide, and pancreatic ductal adenocarcinoma is the most common type. In pancreatic cancer, glycolysis is the primary way energy is produced to maintain the proliferation, invasion, migration, and metastasis of cancer cells, even under normoxia. However, the potential molecular mechanism is still unknown. From this perspective, this review mainly aimed to summarize the current reasonable interpretation of aerobic glycolysis in pancreatic cancer and some of the newest methods for the detection and treatment of pancreatic cancer. More specifically, we reported some biochemical parameters, such as newly developed enzymes and transporters, and further explored their potential as diagnostic biomarkers and therapeutic targets.

## Background

Pancreatic cancer is an extremely aggressive malignant tumor associated with age. It is the fourth leading cause of mortality among all cancers in the US. It is predicted that pancreatic ductal adenocarcinoma (PDAC) will become the second leading cause of cancer-related death among all cancers [1, 2], and morbidity peaks by the age of 80 years old. PDAC is the primary exocrine pancreatic cancer and the most common type of pancreatic cancer, accounting for almost 90% of cases. Mucinous tumors, which are the second most common type of pancreatic cancer, account for less than 10% of cases [1, 2]. Because the majority of pancreatic cancer cases are PDAC, this article mainly discusses glycometabolic rearrangements in PDAC. PDAC classification based on genetic alterations is commonly discussed right now, and researchers count on these subdivided groups, which could provide prognostic measurements for the detection and treatments of PDAC. There are several methods for molecular classification, such as relying on single-genetic marker classification, genomic aberrations, and transcriptomic subtypes. Single-genetic marker classification aims to identify a single gene mutation, and these mutations have either a sufficient rate of occurrence or relate to beneficial treatment decisions [3]. PDAC can be divided into four subgroups according to genomic aberrations: stable genomes (< 50 structural variants per genome), scattered genomes (50–200 structural variants per genome), locally rearranged genomes (> 200 structural variants clustered on < 3 chromosomes), and unstable genomes (> 200 structural variants across the genome); these classifications can be divided into two groups: locally rearranged ones and unstable ones [4]. Transcriptomic subtypes enable unbiased classification and can demonstrate the preferential expression of genes. Based on a multiplatform method including transcriptomic profiles (mRNA and microRNA), whole-genome sequencing, and proteomics studies, a novel clinical subgroup was proposed in 2016. A total of 456 resected PDAC specimens were divided into 4 gene expression-based subgroups: squamous, pancreatic progenitor, immunogenic and aberrantly differentiated endocrine exocrine (ADEX) [5]. Nevertheless, in the following year, a minor mistake was found in this novel method; another similar analysis of 150 resected PDAC samples suggested that the ADEX and immunogenic subgroups of this classification system were derived from nonneoplastic cell contamination [6, 7]. Recently, after analyzing the genomic profiles, transcriptomic profiles, and clinical data of 325 PDAC patients, researchers identified four clinical subgroups: quiescent, glycolytic, cholesterogenic, and mixed [8]. This specific classification system should be explored and researched further because it indicates different clinical outcomes correlated with specific molecular mechanisms.

PDAC is associated with inherited mutations in many genes, including *KRAS, TP53, HIF-1A, PIK3CA, EGFR* and other mutated genes [9–11]. However, the most common driver mutations are in *KRAS* and *TP53*, contributing to more than 90 and 50% of cases, respectively [12]. Mutant genes modulate the expression and behaviors of PDAC, especially those related to metabolism, which is called metabolic reprograming (or rearrangements). *KRAS* stimulates glucose uptake by increasing glucose transporter levels on the cancer cell membrane, which promotes glucose uptake and further increases glycolysis in PDAC [13]; p53, the gene product of *TP53*, was demonstrated to increase glycolysis mediated by *TP53*-induced glycolysis and apoptosis regulator (TIGAR) [14]. Under hypoxic conditions, *KRAS* mutations occur in PDAC cells, which stabilize *HIF1A* and *HIF2A* to regulate pH and glycolysis [15]. The glycometabolic rearrangements occurring in PDAC are the main way energy is produced in PDAC and have been shown to be closely connected with the tumorigenesis of PDAC. Because of the harsh microenvironment and inherited changes of PDAC, PDAC strongly tends to generate energy through aerobic glycolysis, which is also known as the Warburg effect [16, 17].

.The microenvironment is a cellular environment where tumors can exist, grow and invade, and tumors can interact with the surrounding microenvironment [18]. Environmental factors also affect the emergence of pancreatic cancer, and 10% of PDAC patients have a relevant inherited predisposition [1, 19–22]. The dense stroma of pancreatic cancer consists of acellular (fibrin, collagen, fibronectin, and hyaluronan) and cellular (endothelial cells, cancer-associated fibroblasts, nerve cells, pancreatic satellite cells, and immune cells) components that occupy over half the size of the tumor mass. PDAC has an impenetrable desmoplastic stroma and hypovascular microenvironment. Due to the rigid stroma, the vessels are compressed, leading to low perfusion and hypervascularity [23, 24]. This harsh microenvironment provides PDAC with a unique hypoxic and nutrient-deprived environment, which requires PDAC to utilize glucose in an oxygen-deficient manner.

**Fig. 1:**
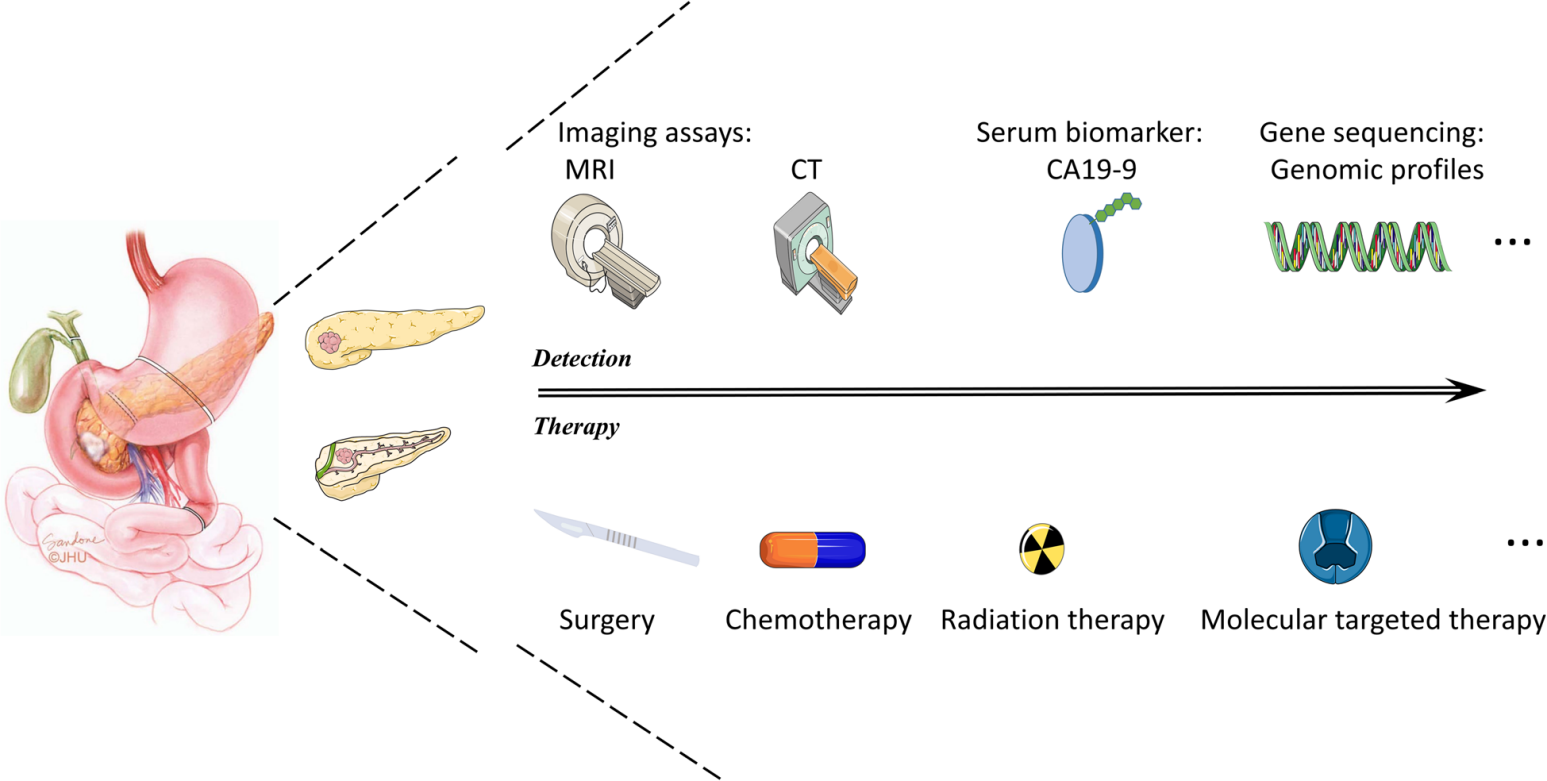
Pancreatic cancer is the most dangerous cancer in the world. The most common form of pancreatic cancer is PDAC, the five-year survival rate of which is 8%. It is difficult to detect PDAC because of the anatomically inaccessible location of the pancreas and difficult to cure PDAC because it is chemo- and radioresistant. Surgery is the only method to achieve a complete cure. Promisingly, olaparib, an inhibitor of PARP, is the newest standard for BRCA1/2-mutant pancreatic cancer patients [25].

Patients with PDAC are generally diagnosed in an advanced stage because they usually do not present any noticeable clinical symptoms in early phases. Thus, approximately 80% of patients with a confirmed diagnosis of PDAC die within a year [22] (Fig. 1). In regard to diagnostic methods, tumor biomarkers exhibit unsatisfactory specificity and sensitivity [26], and computed tomography (CT) examination displays limitations, such as a low resolution for small lesions [20, 22]. Although pathological examination is the gold standard for PDAC diagnosis, its invasiveness restricts its application. Thus, the development of a precise strategy to detect PDAC in an early stage, which will benefit patient prognosis, is urgently needed. Moreover, conventional treatments such as chemotherapy and radiotherapy produce disappointing results, and the indication for surgery, which is the only method to achieve a clinical cure, is quite restricted [27]. Thus, we propose seeking other effective means for PDAC therapy. In conclusion, this article mainly discusses interactions between glycometabolic rearrangements, mainly glycolysis, in PDAC and characterizes factors for early detection of pancreatic cancer as well as therapeutic methods for achieving better clinical outcomes.

## Glycometabolic rearrangements in pancreatic cancer

### Glycolysis

**Fig. 2:**
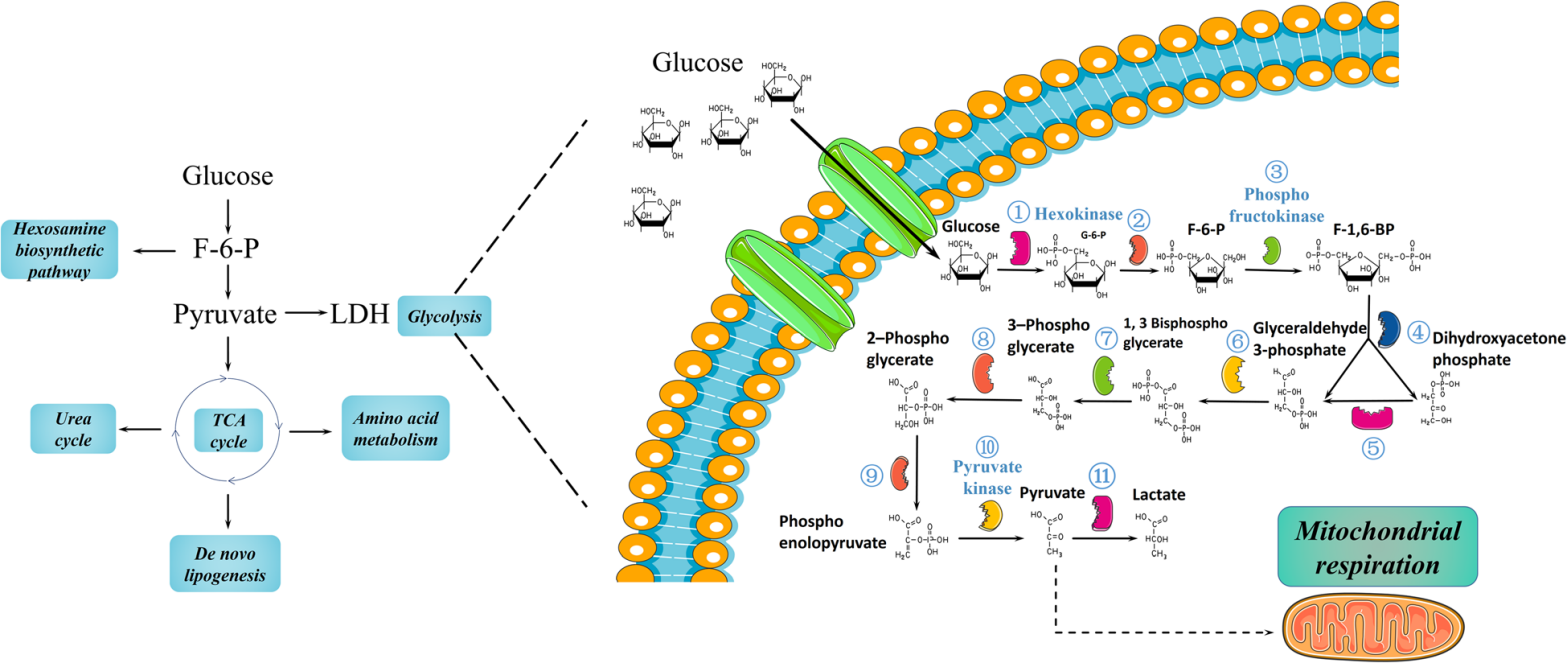
Metabolic reaction in PDAC cells, especially aerobic glycolysis in pancreatic cancer cells. Glycolysis is the main form of energy production in pancreatic cancer, even under normoxia. However, there are also some cancer cells that produce energy through oxidative phosphorylation. GLUT: glucose transporter; G-6-P: glucose-6-phosphate; F-6-P: fructose-6-phosphate; F-1,6-BP: fructose-1,6-biphosphate. 1: Hexokinase; 2: phosphohexose isomerase; 3: phosphofructokinase; 4: aldolase; 5: triose phosphate isomerase; 6: glyceraldehyde 3-phosphate dehydrogenase; 7: phosphoglycerate kinase; 8: phosphoglycerate mutase; 9: enolase; 10: pyruvate kinase 11: lactate dehydrogenase

Carbohydrates are the main ingredient in the daily diet, and cancer applies aerobic glycolysis as a carbon supplement [28, 29]. Glucose is transferred through the cell membrane into the cytoplasm by glucose transporters (GLUTs), including GLUT1, GLUT2, GLUT3, GLUT4, and GLUT5 [30]. GLUT1 and GLUT3 are extensively distributed in all cells as primary transporters to transfer glucose into the cytoplasm [31]. However, most GLUT2 exists in hepatocytes and pancreatic β-cells, and GLUT4 mainly exists in adipocytes and cardiomyocytes, while GLUT5 is distributed in the epithelial cells of the small intestine. Interestingly, GLUT1 is commonly overexpressed in PDAC cells, accompanied by cellular invasiveness induced by the upregulation of matrix metalloproteinase-2 (MMP-2) expression via p53 mutation [32, 33]. Several studies have indicated that MMP-2 overexpression is likely modulated by GLUT1 via the PI3K/AKT and JNK/MEKKI pathways. GLUT1 is transactivated by *KRAS, cMyc*, and HIF-1α but inhibited by p53. Furthermore, GLUT1 is negatively associated with the expression of PTEN in thyroid cancer cells [31, 32]. Similarly, GLUT3 is activated through the IKK/NF-KB axis and inhibited by p53. Due to the correlation between the overexpression of GLUTs and rising uptake of glucose, glucose can be utilized for positron emission tomography (PET) [32, 33]. Glucose transferred into the cytoplasm by a GLUT is catalyzed by hexokinase (HK) and converted into glucose-6-phosphate. Then, phosphohexose isomerase (PGI) converts glucose-6-phosphate into fructose-6-phosphate (F-6-P). F-6-P is catalyzed by phosphofructokinase (PFK) to generate fructose-1,6-biphosphate (F-1,6-BP) [34]. F-1,6-BP is split into 3-phosphoglyceraldehyde (GA3P) and dihydroxy-acetone phosphate (DHAP). Then, the latter phosphotriose is transformed into the former by triose phosphate isomerase (TPI), and 3-phosphoglyceraldehyde is oxidized to 1,3-diphosphoglyceric acid (1,3-DPG) by glyceraldehyde 3-phosphate dehydrogenase (GAPDH) [35] and then catalyzed into 3-phosphoglycerate (3-PG) by phosphoglycerate kinase. 3-PG is converted into 2-PG by phosphoglycerate mutase (PGAM) [36], after which it is dehydrated to form phosphoenolpyruvate (PEP), which is catalyzed by enolase [37]. Following this step, pyruvate kinase catalyzes the transformation of PEP into pyruvate and adenosine triphosphate (ATP) [38]. Then, lactate dehydrogenase (LDH) catalyzes pyruvate conversion into L-lactate, which is the last step of glycolysis [30] (Fig. 2). Glycolysis is the primary metabolic rearrangement in PDAC cells under aerobic conditions, according to Otto Warburg [39].

### Glycolysis and the microenvironment

#### Hypoxic microenvironment

**Fig. 3:**
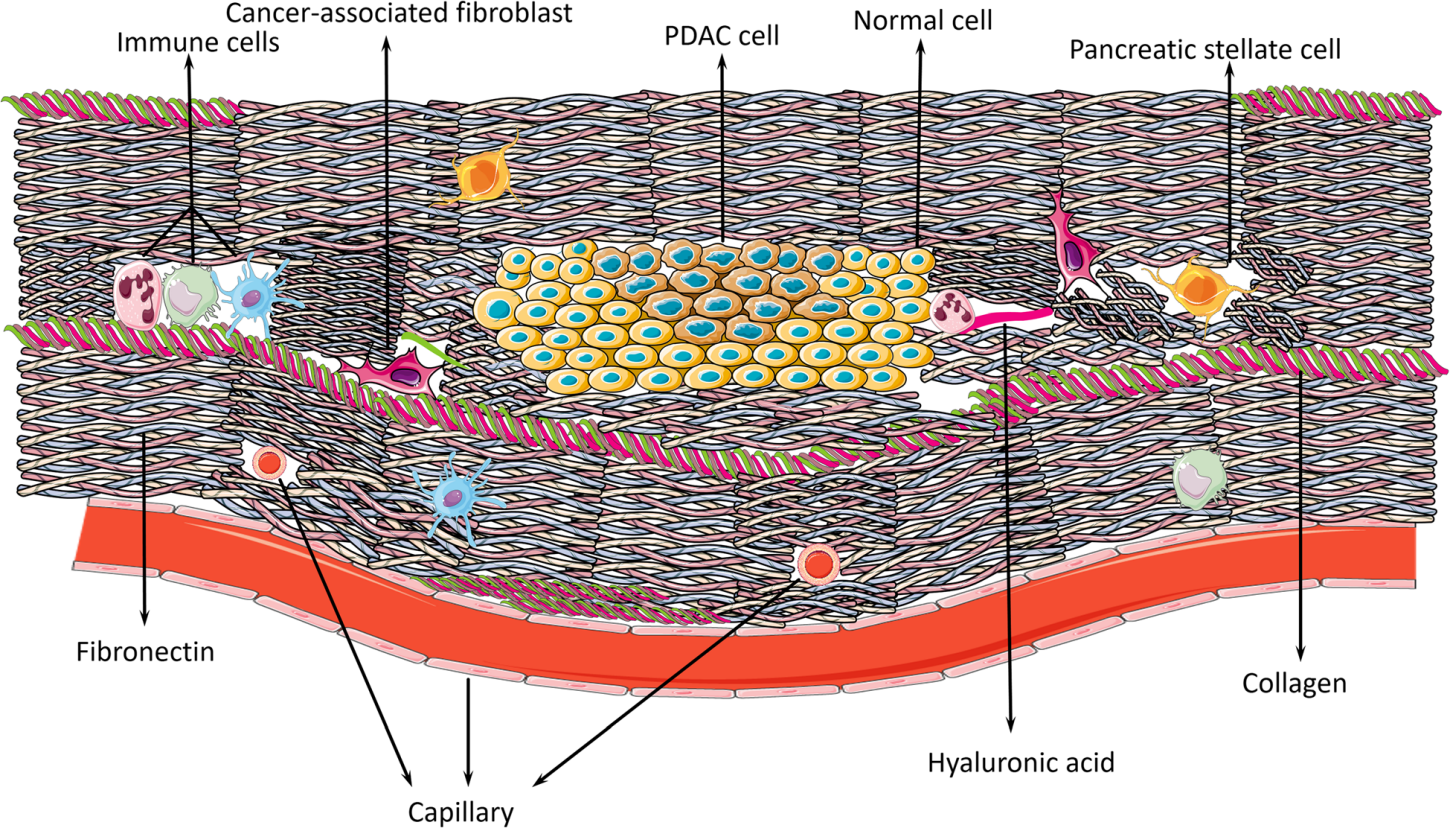
The microenvironment of pancreatic cancer. The stroma consists of cellular components (including immune cells, PSCs, CAFs and cancer cells) and noncellular components (hyaluronic acid, collagen, capillaries and fibronectin). This compact tissue lacking feeding vessels to provide nutrition blocks the diffusion of oxygen and transportation of glucose. In this situation, cells away from the vessels start to convert into glycolytic types. The dense stroma also contributes to chemo/radiotherapy resistance to drugs and resistance to T cells. There is a heated debate on the concrete function of the dense stroma: does it provide more benefits than disadvantages in PDAC? It is worth expending more efforts to unravel this mystery

Aerobic glycolysis means that PDAC cells produce ATP even under aerobic conditions, which is called the Warburg effect, because this process avoids generating reactive oxygen species (ROS). It is a faster way to supply the needs for the biosynthesis of PDAC cells during proliferation, invasion, migration, and metastasis [28]. PDAC as a malignant tumor occurs with universal somatic evolution, which resembles Darwinian processes. Cells that are more suitable for the microenvironment survive in competition, whereas inadaptable cells gradually become extinct. Therefore, what precisely is meant by ‘suitable’? [40] Premalignant PDAC lesions are characterized as the hypovascular type caused by the desmoplastic stroma, and the capillaries in the extracellular matrix (ECM) are basically nonfunctional [41, 42]. In addition, the microcapillary in premalignant lesions is separate from the lesion, such as in carcinoma in situ, and the blood supply, such as a branch of capillary, is encircled by the vascular stroma and separated from the lesion by the basement membrane [40]. The microcapillaries are compressed, making it difficult for them to retract and transport substances. Therefore nutrient substances, such as glucose and oxygen, cannot be transported into tumor tissue directly but instead diffuse from the vessels slowly through the membrane and stroma into tumor cells [43]. This process results in solid tumors that are poorly oxygenated and hypoxic, which might also occur in tumors as a result of fluctuating blood flow [40, 42, 44]. Therefore, the special hypoxic microenvironment facilitates metabolic rearrangements in cancers to make the best use of the available oxygen when the oxygen level is quite low, such as in PDAC [45] (Fig. 3).

Organisms need oxygen to sustain energy production. When oxygen is lacking, it causes immense stress to cells. Hypoxia-inducible factor (HIF) has been widely found to be expressed at elevated levels under hypoxia to match the oxygen supply [46–48]. HIF-1α is unstable under normoxic conditions due to immediate proteasomal degradation [49]. However, under hypoxia, HIF-1α and HIF-2α are both stable [50, 51]. HIF-1α is extensively expressed in all cells; however, HIF-2α is restricted to only several cell types, including the vascular endothelium, hepatocytes, and kidney epithelial cells [52]. HIF-1α is transported into the nucleus and binds to HIF-1β (heterodimeric counterpart), integrating with the coactivators CBP and p300 to generate activated HIF-1 combined with hypoxia-responsive elements as a complex to regulate hypoxia-related gene expression [53, 54]. Additionally, the overexpression of HIF-1α was proven to be connected with a poor prognosis in several cancers in a study [55]. The authors discovered that HIF-1α regulates glucose metabolism-related rearrangements in PDAC and that the expression of HIF-1α in tumor tissue is higher than that in surrounding normal tissues even under normoxic conditions [55]. Upregulation of HIF-1α expression has been associated with relatively early relapse and shortened overall survival (OS) [56, 57]. The poor clinical outcomes associated with HIF-1α may be related to epithelial-to-mesenchymal transition (EMT), producing essential effects on the invasion and metastasis of PDAC. EMT is a transition from an epithelial phenotype to a mesenchymal phenotype. This process is accompanied by the loss of epithelial biomarkers, such as E-cadherin, desmoplankins, claudins, and cytokeratins, and the gain of mesenchymal biomarkers, such as N-cadherin, vimentin, and fibronectin. E-cadherin, as a glycoprotein, can boost adhesion among cells and maintain cytoskeletal organization, and loss of E-cadherin leads to decreased cell adhesiveness and promotes EMT and metastasis [58]. Together with increasing biomarker levels in EMT, transcriptional repressors of E-cadherin are also highly expressed, such as zinc-finger E-box binding homeobox (ZEB), SNAIL1 (SNAIL), SNAIL2 (SLUG), TWIST, and FOXC2, which are regulated by the hypoxic status to suppress E-cadherin expression for EMT initiation [59]. The process of EMT involves several pathways actually modulated by HIFs under hypoxia. First, HIF-1α mediates the TGF-β1-induced pathway, synergistically activating the transcription factors SNAIL, ZEB, and TWIST in a Smad-dependent manner, which leads to suppression of E-cadherin and regulation of TGF-β target genes [60]. Another pathway involved in the hypoxia-EMT process is the Notch signaling pathway. The Notch pathway enhances SNAIL expression in cooperation with HIF-1α under hypoxia via two synergistic manners: directly upregulating SNAIL expression through intracellular Notch binding to the SNAIL promoter and indirectly upregulating SNAIL expression through intracellular Notch recruitment to the LOX promoter to enhance LOX and SNAIL production [61]. Studies have suggested that the PI3K/AKT pathway is also involved in the EMT process related to HIF-1α under hypoxia. In hepatocellular carcinoma, activated HIF-1α is indicated to be a downstream regulator of the PI3K/AKT pathway and is accompanied by differences in EMT biomarkers [62]. The other signaling pathway related to hypoxia-EMT is the Wnt/β-catenin signaling cascade. HIF-2α induces the Wnt/β-catenin pathway in PDAC, which then mediates the activation of SNAIL, inhibiting E-cadherin production to induce the EMT process [63]. Nuclear factor-κB (NF-κB) is an omnipresent transcription factor in cancer cells involved in many cellular actions, including EMT. NF-κB activates the transcriptional repressors TWIST and SNAIL in a HIF-dependent manner, which inhibits the expression of E-cadherin and increases the expression of N-cadherin and vimentin, indicating the activation of EMT [51, 64]. Recently, some studies have established the importance of the hypoxic microenvironment and EMT; immune and inflammatory cells and their secreted factors in the microenvironment have tight relationships with the formation of EMT. For instance, tumor necrosis factor-α (TNF-α) secreted by tumor-associated macrophages induces activation of the SNAIL promoter and promotes the EMT process [65, 66].

Overexpressed HIF-1α fuels the expression and activity of GLUTs, HK, PFK-L, ALD-A, GAPDH, PGAM-B, ENO-α, and PKM-2, which increase glycolysis and glucose uptake in PDAC [67, 68]. In a hypoxic microenvironment, mutant *KRAS* stabilizes *HIF1A* and *HIF2A* to augment the expression of carbonic anhydrase 9 to regulate pH and glycolysis in PDAC [15]. Moreover, transforming growth factor beta-induced (TGFBI) boosts the focal adhesion kinase (FAK) signaling pathway by binding to the integrin αVβ5, a cell membrane protein that stabilizes HIF-1α and then facilitates glycolysis in PDAC [69]. Recently, chromatin immunoprecipitation assays were applied to detect the occupancy of HIF-1α on the *CTPS1* and *TKT* gene promoters. In human PDAC, CA IX (a HIF-1α activity symbol) was colocalized with *TKT* and *CTPS*. HIF-1α stabilized by MUC1, a highly expressed transmembrane protein, could modulate the expression of *TKT*, *CTPS1*, and other genes related to glycolysis, leading to gemcitabine and FOLFIRINOX resistance and increasing glycolysis in PDAC [70]. After knocking down MUC1 expression, reductions in HIF-1α protein expression, glucose uptake, and lactate release were observed [71]. Another study demonstrated that MUC1 facilitated HIF-1α translocation into the nucleus to act as a transcription factor for genes responsible for PDAC proliferation and invasion [72]. Moreover, the colocalization of the HIF-1α protein and VEGF mRNA identified via immunohistochemical analysis suggested that HIF-1α mediates the transcription of VEGF to induce angiogenesis in PDAC [73]. On the one hand, receptor for advanced glycation end products (RAGE) binding to *KRAS* fuels the activation of HIF-1α and promotes tumor development and glycolysis in PDAC under hypoxia. On the other hand, hypoxia induces HIF-1α-independent RAGE expression, which in turn activates *KRAS* signaling pathways (*RAF-MEK-ERK* and *PI3K-AKT*), stabilizing and activating HIF-1α [74]. Furthermore, the expression of miR-125a was shown to be significantly increased when HIF-1α expression was knocked down in the PANC-1 cell line, and HIF-1α was found to negatively regulate miR-125a, which plays vital roles in apoptosis, invasion, and metabolic rearrangements [75]. The mutant gene profiles in addition to hypoxic and hypovascular conditions, especially with the regulation of HIF-1α, contribute to the distinctive metabolic rearrangements related to aerobic glycolysis through multiple intersecting molecular pathways. Interestingly, upregulated glycolysis in PDAC can promote EMT phenotypes in PDAC cells by maintaining low ROS levels [76], which suggests a direction for inhibiting PDAC metastasis.

Given the increasing experimental information available, the stroma and tumor microenvironment (TME) are now considered to exert significant effects on PDAC metabolic rearrangements, carcinogenesis, metastasis, and resistance to radio/chemotherapy, suggesting a poor prognosis [24, 41]. Hypoxia and the hypovascular microenvironment cause tumor cell glycolysis and hinder immune cell infiltration and drug penetration, leading to immunosuppression and chemoresistance [77]. In this situation, the stiff stroma is also quite difficult for oxygen and glucose to pass through [78]. Recently, it was demonstrated that SerpinB2, which regulates CAF interactions and engagement with collagen in the matrix and is required for normal collagen remodeling associated with local invasion, is lost in pancreatic cancer [41]. However, there is a heated dispute over the contradictory functions of the dense stroma in PDAC carcinogenesis. Depleting the stroma in PDAC mice by degrading HA with enzymes or an inhibitor of Sonic Hedgehog (IPI926) restrains the development of PDAC [79, 80]. However, some clinical data have presented different conclusions, finding that the stiff stroma restrains the development of pancreatic cancer rather than facilitates progression due to the limited space [81]. The concrete function and molecular mechanisms of the stiff stroma in PDAC are still unclear, but the stroma is tightly correlated with aerobic glycolysis.

#### Pancreatic stellate cells and the stroma

Glycolysis emerges under microenvironmental selection [44], and it can produce energy for proliferation, invasion, migration, and even metastasis in the hypoxic state regulated by HIF-1α [70]. However, this process is quite deficient, accounting for only 31% of aerobic respiration [82]. Interestingly, with further research, a new theory called the ‘reverse Warburg effect’ was recently developed by studying metastatic breast cancer, which claims that metabolic reprogramming occurs in cancer-associated fibroblasts (CAFs), which also undergo aerobic glycolysis [83]. Nonetheless, anabolic products, such as pyruvate and L-lactate, can be transferred into cancer cells through mono-carboxylate transporters 4 (MCT4) and mono-carboxylate transporters 1 (MCT1) to the mitochondria for oxidative phosphorylation [84, 85]. L-lactate can be converted into pyruvate by LDHB in cancer cells [84]. ROS generated by the mitochondria in cancer cells diffuse into CAFs, inducing an increase in glycolysis regulated by upregulated HIF-1α expression [23]. This process is a more efficient way for tumors to produce energy than aerobic glycolysis [82]. In summary, the reverse Warburg and Warburg effects demonstrate the great plasticity and flexibility of cancer glycometabolic rearrangements. This inspired us to develop diverse therapeutic strategies, such as targeting MCT and suppressing glycolysis, addressing both pathways to inhibit the growth and invasion of PDAC [84, 85]. The reverse Warburg effect is considered to be similar to the Warburg effect because increased glycolysis occurs under normoxia in tumors [85].

**Fig. 4:**
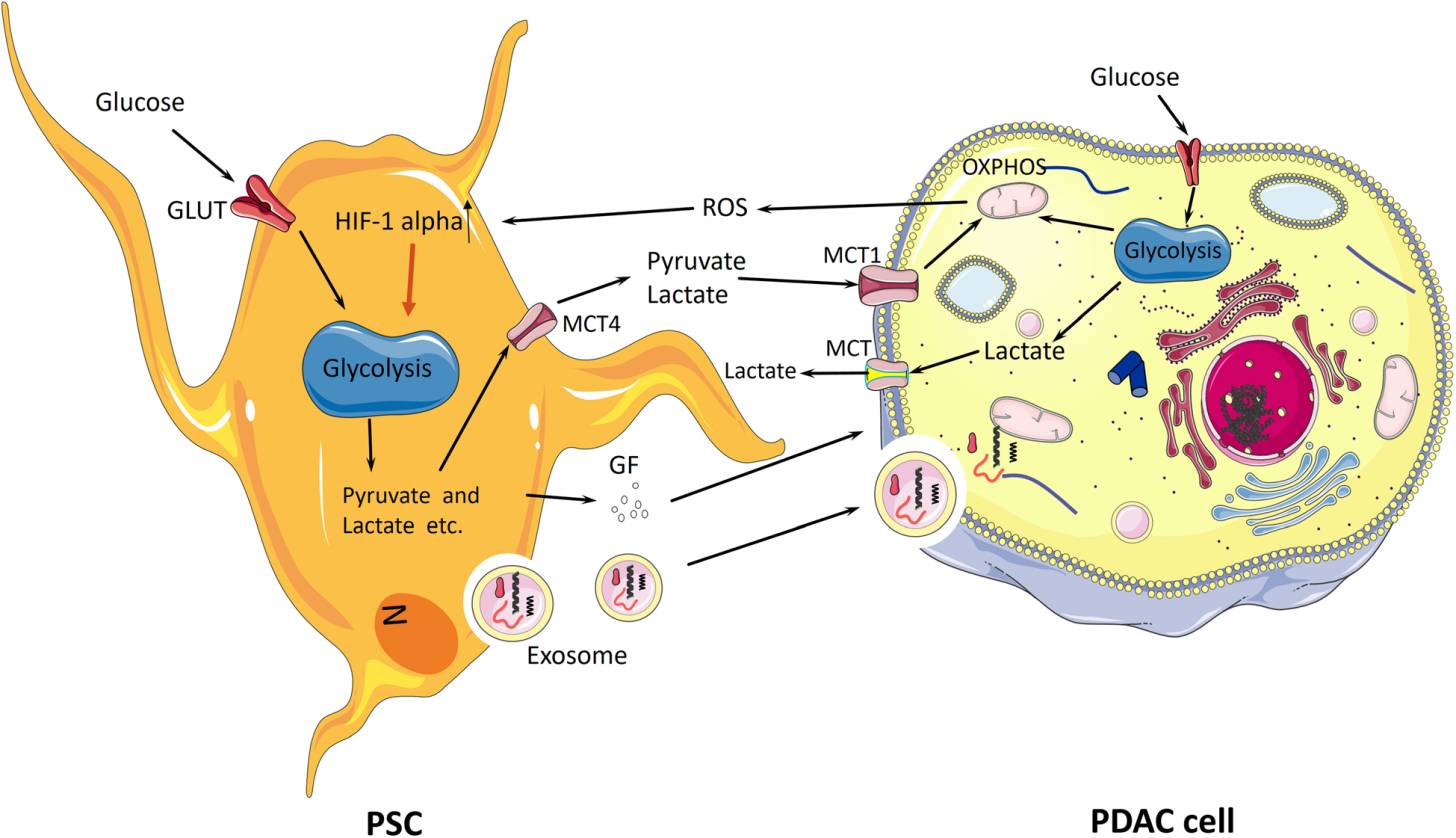
Interaction between a PSC and PDAC cell. Reverse Warburg effect: PSCs utilize glucose to conduct glycolysis and produce pyruvate and lactate, which can enter PDAC cells and undergo mitochondrial respiration to produce energy. The metabolites of glycolysis in PSCs can be transported into pancreatic cancer cells for oxidative phosphorylation via MCT-4 and MCT-1. ROS generated by pancreatic cancer cells then spread into PSCs, inducing an increase in HIF-1α expression to further augment glycolysis in the PSCs. Exosomes secreted by PSCs containing gene products and lipids are internalized by pancreatic cancer cells. This interaction plays a vital role in the progression of pancreatic cancer and contributes to immune escape

.Pancreatic stellate cells (PSCs), first isolated and cultured in 1998 [86], are the fundamental constituent of the TME and the most significant type of cancer-associated fibroblast (CAF) that induces the underlying molecular mechanisms of pancreatic cancer [87]. Under normal conditions, PSCs are localized in the basolateral aspect of pancreatic acinar cells as quiescent pancreatic stellate cells (qPSCs), generating ECM proteins and relevant enzymes [88]. They are reprogrammed into activated pancreatic satellite cells (aPSCs) in PDAC by environmental stress, molecular pathways, risk factors, and cellular factors, leading to the accumulation of abundant ECM proteins and a desmoplastic stroma [89]. In PDAC, to sustain biosynthesis for tumor growth and proliferation, PSCs and PDAC cells highly utilize glucose via the Warburg effect and reverse Warburg effect [23]. The complete interactions between PDAC cells and PSCs remain to be explored. However, some data indicate that Zeb1 in PSCs is responsible for *KRAS* activation in PSCs [90]. Coincidently, the oncogene *KRAS* mutates, causing enhanced glucose uptake and activating pivotal enzymes in glycolysis in both PDAC cells and PSCs. Furthermore, mutant *KRAS* signaling also promotes Sonic Hedgehog (SHH) secretion by PDAC cells, which then facilitates the expression of GAS6, IGF1, GM-CSF and other cytokines in PSCs [91]. This progress is responsible for feedback signaling via the IGF1R/AXL axis, which induces the phosphorylation of PI3K/AKT, increasing the mitochondrial respiration capability of PDAC cells. Additionally, inhibiting the ERK1/2 pathway in PSCs can suppress PDAC cell-stroma interactions and metabolic rearrangements [78]. Interestingly, exosomes secreted by PSCs containing miRNAs, mRNAs and metabolites, such as lactate and amino acids, also lead to the growth of PDAC cells through the exosomal transport of contents, such as miR-21, miR-1246 and miR-1290, which are responsible for the progression of PDAC [92–94]. Furthermore, the metabolites in exosomes can enter PDAC cells and provide supplements for oxidative phosphorylation to increase energy production in the mitochondria. MiR-210 can also modulate the interplay between PDAC cells and PSCs [95]. PSCs have effects on PDAC cells, such as metabolic rewiring, microenvironmental homeostasis maintenance, immunosuppression and immune evasion to regulate PDAC survival and metastasis (Fig. 4). These interactions between PDAC cells and PSCs offer researchers novel strategies to disrupt the pancreatic stroma to suppress the growth of PDAC and provide an accurate target for PDAC therapy.

### Metabolites and oxidative phosphorylation

As continuous upregulation of aerobic glycolysis occurs in PDAC cells under hypoxia/anoxia [45], lactic acid generated from the process of glycolysis causes enormous extracellular and intracellular pH changes caused by hydrogen ions [40]. Normal and stromal tissues will be affected by the acidic microenvironment. Apoptosis and autophagy are induced by acidosis in healthy cells, and the structure of the stromal tissue is modified [96, 97]. However, PDAC cells can survive in this microenvironment due to some adaptive mutations, such as mutations in *TP53* and *KRAS*. The acidic anaerobic microenvironment is a selective pressure that allows most malignant tumor cells to survive in competition for finite substrates and living areas with normal cells, as well as to migrate, invade and metastasize more easily [19, 28, 40, 98].

Interestingly, accumulating literature has demonstrated that oxidative phosphorylation (OXPHOS) is upregulated in some glycolytic cancers, including PDAC, which might be driven by the oncogene *KRAS* and the loss of *LKB1* [99]. According to the reverse Warburg effect, PSCs and CAFs undergo glycolysis and transport metabolites into PDAC cells that are used for mitochondrial respiration to generate energy [84]. Meanwhile, pancreatic cancer stem cells rely on mitochondrial OXPHOS, which may be correlated with the suppression of MYC and the MYC/PGC-1α ratio, so mitochondrial agents and genetic therapy can easily target this phenotype [31]. Nonetheless, some studies have revealed that OXPHOS is not consistently suppressed. Instead, it can be reactivated under some conditions, such as activation of the *PI3K-AKT-mTOR* and *LKB1-AMPK-p53* pathways [99]. As accumulating lactate is released into the microenvironment, glycolysis may be affected by the acidic microenvironment. When glycolysis is inhibited, glycolytic PDAC cells transport pyruvate into the mitochondria for OXPHOS, as the acidified microenvironment makes the reprogrammed microenvironment transport glucose and oxygen more efficiently and easily, which may also be associated with PDAC progression [100]. Intriguingly, mitochondrial OXPHOS accounts for more than 70% of the overall ATP production in cervical and breast cancer cell lines under normoxia, but it is reduced to less than 40% under hypoxic conditions [99]. However, the concrete relationship between OXPHOS and PDAC progression is still unclear, and the specific molecular mechanisms are under investigation. Although the mechanisms are unclear, some data verify that OXPHOS inhibitors can serve as promising therapeutic agents in PDAC [101]. Overall, proliferative PDAC acts as a glycolysis-dominant metabolic cancer with a possible alternative OXPHOS pathway being activated when glycolysis is inhibited. These two metabolic pathways render PDAC aggressive, allowing it to adapt to different microenvironmental conditions. Targeting or inhibiting OXPHOS should be thoroughly considered and verified because the plastic reprogramming of PDAC metabolism may switch OXPHOS cells into more aggressive glycolytic cells.

Immune cells in the cancer microenvironment, including T cells, B cells, natural killer cells, dendritic cells, neutrophils, and macrophages, should convert into glycolytic types to adapt to increasing biosynthetic needs for their anabolic functions and rapid growth in activated states [102–106]. The secretion of lactate and depletion of glucose by cancer cells can inhibit the functions of immune cells, and PDAC cells are able to escape the immune response [107, 108]. Mechanistically, MCTs exist in stromal cells, such as PSCs and immune cells, as mentioned before; to sustain ceaseless glycolysis in PDAC, the lactate and H^+^ released into the extracellular stroma are transported into immune cells, which induces intracellular acidification and inhibits glycolysis, ultimately resulting in functional damage to the immune cells [109]. Moreover, the lactate levels in PDAC are not as high as we hypothesized, possibly because the abundant CAFs and PSCs as well as immune cells utilize lactate via conversion into pyruvate for OXPHOS [110]. However, regulatory T cells with distinctly lower glycolytic characteristics increase resistance to the low-glucose, high-lactate tumor milieu and retain immunosuppressive functions that may be related to peripheral tolerance in the low-glucose, high-lactate tissue environment [111]. Based on the negative association between glycolysis and immune cells, a novel metabolism-tumor-stroma (MeTS) score was proposed to guide therapy selection for different metabolic classifications. There are four different type: MeTS1, OXPHOS tumors with a high T cell proportion, also called “hot”; MeTS2, reverse Warburg tumor cells with OXPHOS tumor cells and glycolytic stromal cells; MeTS3, a mixed classification having both OXPHOS and glycolytic cells; and MeTS4, glycolytic cancers with a low T cell proportion, also called “cold” [112]. However, not all solid tumors are identified as a specific glycolytic type because of tumor heterogeneity. Furthermore, even in a given tumor, not all cancer cells have the same metabolic identity. PDAC cells have significant glycolytic metabolism features with the reverse Warburg effect observed in PSCs and OXPHOS occurring in PDAC cells, so PDAC should be sorted as MeTS2 or MeTS3 depending on individual characteristics, according to the sorting method.

### Clinical subgroups and glycolysis

New publications classify PDAC into 4 subtypes: quiescent, glycolytic, cholesterogenic, and mixed according to metabolic reprogramming features. Among the subtypes, the glycolytic subtype with high expression of glycolysis-related genes exhibits glycolysis-predominant metabolism with the worst clinical outcomes, and the cholesterogenic subtype with high expression of sterol biosynthesis- or cholesterogenesis-related genes promotes pyruvate entry into the mitochondria through mitochondrial pyruvate carriers (MPCs) to produce acetyl CoA for cholesterol biosynthesis and has the best clinical outcomes [113]. The quiescent and mixed subtypes with low and high expression of both metabolic pathways, respectively, show intermediate clinical outcomes. Mechanistically, the glycolytic subtype has a tight correlation with the oncogene *KRAS* and a weak correlation with *MYC.* Moreover, *HIF1A* and related genes, such as *LDHA* and *SLC16A3,* exhibit upregulated expression in the glycolytic subtype, which may indicate aggressive features, and the expression of the transcriptional regulator sterol regulatory element binding transcription factor 2 (*SREBF2*) is upregulated in the cholesterogenic subtype [8]. *SREBF2* was proven to attenuate the activation of the 3-hydroxy-3-methylglutaryl coenzyme A reductase (HMGCR) promoter, which is upregulated in pancreatic intraepithelial neoplasia driven by KRAS^G12D^ and may be associated with relatively good outcomes [114].

### Pivotal enzymes in glycolysis

Intriguingly, almost every enzyme in glycolysis plays a dual-function role during the progression of pancreatic cancer. The enzymes usually perform their catalytic activity in the cytoplasm and regulate transcription factors in the nucleus. The two processes are both crucial in the proliferation, invasion, migration, and metastasis of PDAC, and some new tactics for therapy and detection of PDAC may be developed from this information.

Glucose is converted into glucose-6-phosphate (G-6-P) by HK, which is the first step of glycolysis and rate limiting. HK has four isoenzymes, and glucokinase is the fourth type of HK that mainly exists in the liver and pancreatic β-cells [115]. The product, G-6-P, and long-chain fatty-acyl-coA inhibit the activation of HK. HK2 binds to the outer mitochondrial membrane through voltage-dependent anion channels (VDACs), and HK2 binding to the mitochondria increases the glycolytic capacity and promotes the immortalization of PDAC [27]. Interestingly, the upregulation of HK2 expression is mediated by HIF-1α, as mentioned before. Recently, *PTEN/p53*-deficient prostate cancer cells were found to exhibit increased expression of HK2, in which PTEN loss activated the AKT-mTORC1-4EBP1 axis to increase HK2 mRNA translation and loss of *p53* inhibited miR-143 biogenesis to enhance HK2 mRNA stability. Moreover, miR-34a was shown to directly target HK1 and HK2, which suppressed glycolysis and promoted mitochondrial respiration [35]. Currently, KRAS4A, the unique palmitoylation-depalmitoylation cycle of the RAS isoform, is known to colocalize with HK1 on the outer mitochondrial membrane, and HK1, as a downstream effector of KRAS4A, can enhance glycolytic flux and cancer progression. Intriguingly, KRAS4A displays twice the effect of KRAS4B at the same gene expression level. When HK1 is silenced, however, the difference disappears. This indicates that KRAS4A directly binds to HK1 and acts downstream of KRAS4A to mediate transcription, but palmitoylated KRAS4A inhibits binding with HK1 [116]. Based on the mechanisms linking KRAS4A and HK, targeting KRAS4A to reduce HK levels should be thoroughly studied in clinical research. The expression of HK is linked to increasing glycolysis and an unfavorable clinical outcome in PDAC. 2-Deoxy-D-glucose (2-DG) is an analog of glucose that has recently been used as an HK inhibitor because it can be phosphorylated by HKs but not catalyzed further [117]. Another small molecule, 3-bromopyruvate (3-BP), and the novel agent methyl jasmonate (MJ) can suppress glycolysis and cancer growth in cancer patients [118, 119]. It was concluded that the lncRNA hox transcript antisense RNA (HOTAIR) is tightly linked with HK2 in PDAC patients. Furthermore, HOTAIR promotes the expression of HK2, and HOTAIR and HK2 are overexpressed in both the serum and tumor tissues of PDAC patients [120]. Therefore, their detection may indicate PDAC progression, and targeting HOTAIR to reduce HK2 expression may lead to the discovery of novel therapeutic strategies for PDAC.

Phosphofructokinase (PFK), which catalyzes the second committed step of glycolysis that involves the conversion of F-6-P into F-1,6-BP, is activated by fructose-2,6-biphosphate (F-2,6-BP), which is the strongest allosteric activator of all (including but not limited to ADP, AMP, F-1,6-BP, and F-2,6-BP), and is inhibited by high concentrations of ATP and citrate [28, 34, 121]. Interestingly, F-2,6-BP is catalyzed and hydrolyzed by a dual-function kinase that has two separate catalytic centers, phosphofructo-2-kinase/fructose-2,6-biphosphatase (PFKFBs) [35]. More precisely, the level of PFKFB3 but not that of other PFKFBs, as a target of HIF-1α that displays the highest phosphofructo-2-kinase activity, is significantly elevated in aggressive cancers, such as PDAC, colon cancer, and breast carcinoma [27, 122]. Similarly, PFKFB4 was also found to have a homologous role in PDAC. PFKFB3 and PFKFB4 are both induced by HIF-1α, hypoxia and augmented expression of the *GLUT* and *VEGF* genes [122]. The glucagon-mediated activation of cAMP-dependent protein kinase (AMPK) is responsible for phosphorylation and inducing attenuation of the phosphofructo-2-kinase activity and augmentation of fructose-2,6-biphosphatase activity in PFKFB3 [123]. Additionally, PFKFB3 is located in both the cytoplasm and nucleus, suggesting that it correlates with the proliferation and invasion of PDAC via transcriptional regulatory effects in addition to glycolytic catalysis, similar to other enzymes (pyruvate kinase) in glycolysis. Nuclear localization of PFKFB3 is correlated with enhanced expression of vital cycle proteins, such as cyclin-dependent kinase-1, cyclin-dependent-25c, and cyclin D3, and reduced expression of the cell cycle inhibitor p27 [124]. These specific characteristics of PFKFB3 can be inhibited by 3-(3-pyridinyl)-1-(4-pyridinyl)-2-propen-1-one (also known as 3-PO) to downregulate the synthesis of F-2,6-BP and reduce glycolytic flux [125]. It has been suggested that inhibiting the HIF-1α/PFKFB3/PFK-1 axis with metformin could suppress glycolysis and impair cancer growth in hepatocellular carcinoma, but whether similar effects can be achieved in other cancers is not yet clear [126]. Recently, miR-135 expression was shown to be elevated in PDAC as a result of glutamine deprivation. This upregulation is dependent on glutamine deficiency and mutant *p53* activated by ROS, which directly promotes miR-135 expression. MiR-135 directly targets PFK expression and leads to decreased mRNA and protein levels, thereby suppressing aerobic glycolysis and increasing OXPHOS flux for attenuation of glutamine dependence to promote PDAC survival in a low-glutamine environment [127]. This information indicates that suppressing miR-135 in the context of enhanced PFK expression and increased glycolysis in PDAC still represses tumor growth. However, we hypothesize that the novel strategy of targeting miR-135 in combination with inhibitors of glutaminase (constructing a glutamine-deficient condition) may create a promising treatment for aggressive PDAC. Similarly, PFK-1 is O-GlcNAcylated at serine 529, which inhibits F-2,6-BP binding with PFK-1 to suppress PFK-1 activity under hypoxia. With reduced PFK-1 activity and glycolytic flux, cancer cells redirect glucose from glycolysis to the pentose phosphate pathway, which shuttles glucose into pathways for protein and DNA synthesis. Conversely, blocking PFK-1 glycosylation at serine 529 impairs cancer formation and proliferation in vivo and in vitro [128].

Notably, pyruvate kinase (PK) catalyzes the conversion of phosphoenolpyruvate into pyruvate and is the last rate-limiting step in glycolysis [35]. F-1,6-BP acts as an allosteric activator of pyruvate kinase. The allosteric inhibitors of pyruvate kinase include acetyl-CoA, ATP, and long-chain fatty acids. Posttranslational modification (PTM) of pyruvate kinase can also regulate its activity. Pyruvate kinase consists of four isoforms: *PKLR* encodes PKL and PKR; *PKM* encodes PKM-1 and PKM-2 via a relatively opposite swapped splicing mechanism. PKL is present in the kidneys and healthy liver, PKR exists in erythrocytes, PKM-1 is abundant in differentiated somatic cells, such as brain and muscle cells, and PKM-2 is expressed in fetal tissue and proliferating cells. A previous study showed that PKM-2 is the central kinase in cancer cells in a dimeric form and has no catalytic affinity for phosphoenolpyruvate [30]. In this situation, there are increasing levels of glycolytic intermediates that could be utilized as precursors for metabolic biosynthesis (such as synthesis pathways for lipids, amino acids, and nucleotides), which could perfectly meet the changing growth requirements in PDAC. Mechanistically, PKM-2 catalyzes a reaction in glycolysis and regulates gene transcription related to proliferation and invasion in PDAC, and it has been demonstrated that high polypyrimidine tract binding protein expression promotes *PKM* splicing to confer drug (gemcitabine) resistance to PDAC cells [38]. With the transformation of PKM-2 from a dimer to a tetramer, PKM-2 has a high affinity for phosphoenolpyruvate, producing a low concentration of metabolic precursors. This transition can be activated by thieno [3,2-b] pyrrole [3,2-d] pyridazinone (TEPP-46), which activates PKM-2 and impairs tumor growth and proliferation, revealing obvious antitumor activity [129]. In addition, PKM-2 induces gemcitabine resistance by downregulating p38 mitogen–activated protein kinase activity, and silencing PKM-2 strongly enhances gemcitabine-resistant cell apoptosis [130].

The lactate dehydrogenase (LDH)-mediated catalysis of pyruvate into lactate is significantly increased in PDAC cells. Previously, the expression of LDH-A (also called LDH-M and encoded by *LDHA*), which catalyzes the conversion of pyruvate into lactate during the last step in glycolysis, was found to be increased in PDAC and other aggressive tumors. However, the levels of LDH-B (also known as LDH-H and encoded by *LDHB*), which preferentially performs the reversible conversion of lactate and pyruvate, were also shown to be increased in PDAC [121, 131]. Nevertheless, there is still an argument to be made for targeting LDH-B, which suppresses the glycolytic subtype of PDAC cells and inhibits the proliferation, invasion, and migration of PDAC [132]. Moreover, a clinical study noted that LDH-A in the serum was associated with a poor prognosis after surgery, which might be correlated with a relatively low pH facilitating tumor relapse, as mentioned above (acidosis selects cells that can survive in that microenvironment and leads to mutations, and low pH stimulates tumor invasion and migration) [19, 23, 24]. Another study reported that Forkhead box protein M1 (FOXM1) promotes glycolysis, glucose consumption, and lactate production in PDAC by upregulating *LDHA* gene expression and augmenting LDHA activity. Unsurprisingly, the FOXM1/LDH-A pathway is also responsible for the progression and growth of PDAC [39]. Targeting LDH-A with siRNA and small molecule inhibitors leads to decreased tumor growth. The selective inhibitor 3-dihydroxy-6-methyl-7-(phenylmethyl)-4-propylnaphthalene-1-carboxylic acid (FX-11) impairs the progression of lymphoma and PDAC xenografts in combination with FK866, a synthetic NAD^+^ inhibitor [133]. Recently, FX-11 in combination with TEPP-46 was employed to treat PDAC cells and demonstrated augmented antitumor activity without obvious toxicity. A phase III trial evaluating the combined treatment has been completed, and the combination is a promising tactic for PDAC therapy [129]. It has been shown that acetylation of LDH-A at lysine 5 (K5) occurs. Acetylation inhibits LDH-A activity, reduces protein levels and promotes the degradation of LDH-A through chaperone-regulated autophagy, which is related to low-efficiency glycolysis by blocking the conversion of pyruvate into lactate, impairing the growth and progression of tumors. Intriguingly, LDH-A strongly accumulates in PDAC, which is accompanied by strongly decreased LDH-A K5 acetylation [134]. Resuming or accelerating the acetylation of K5 in LDH-A in PDAC may produce promising therapeutic results. Novel *N*-hydroxyindole-based (NHI) inhibitors targeting LDH-A impair proliferation, growth, and migration in PDAC. Moreover, when an NHI inhibitor is synergistically cultured with gemcitabine, it shows enhanced anticancer activity against PDAC [135]. Krüppel-like factor 4 (KLF4), a zinc-finger transcription factor, negatively regulates the transcriptional activity of LDH-A, thus impacting glycolysis and tumorigenesis in PDAC. The KLF4/LDH-A axis has close correlations with glycolysis and the progression of PDAC, as evidenced by both clinical data and experimental data [136].

Aldolase is an enzyme in aerobic glycolysis, and aldolase gene expression is significantly elevated in pancreatic cancer. Aldolase includes three isozymes, aldolase A, aldolase B, and aldolase C, encoded by three different genes, *ALDOA, ALDOB,* and *ALDOC,* respectively. Aldolase A is present in muscle tissues; aldolase B is extensively expressed in the kidneys, liver, stomach, and intestine; and aldolase C is expressed in the brain. Remarkably, aldolase A is highly relevant to various malignant cancers, including PDAC, and its expression is significantly increased in metastatic PDAC, in which it is used as a crucial indicator for detection. After treatment with transforming growth factor-β, PANC-1 cells exhibit increased *ALDOA* expression, leading to enhanced glycolysis. Furthermore, silencing aldolase A decreases aerobic glycolysis and ROS generation and inhibits the proliferation and metastasis (as indicated by EMT markers such as E-cadherin, N-cadherin, and vimentin) of PDAC in vivo and in vitro [137]. Similar to other enzymes involved in glycolysis, aldolase A is speculated to play dual-function roles in PDAC: catalysis in the cytoplasm and transcriptional regulation in the nucleus, which has been clarified in the pathogen Francisella [138]. A study showed that aldolase A could be inhibited by the hypoxic cytotoxin 3-[2-hydroxyethyl (methyl)amino]-2-quinoxalinecarbonitrile 1,4-dioxide (TX-2098). TX-2098 treatment suppressed the expression of HIF-1α, vascular endothelial cell growth factor, GLUT1, and aldolase A, leading to distinct antitumor efficacy in a xenograft PDAC model [139]. Moreover, naphthalene-2,6-diyl bisphosphate (ND1) is an active site substrate mimic that acts as an effective inhibitor of aldolase A, but it can be hydrolyzed easily. To address this shortcoming, a series of analogs of ND1 were extensively researched to identify covalent and noncovalent inhibitors. The most stable noncovalent inhibitor identified is NDB, which has two difluoromethylene insertions that do not impair the binding affinity. However, biochemical assays showed that methylene insertion weakened the effect of ND1 [140]. It is clear that the lncRNA DIO3OS promotes the growth of PDAC tumors with low expression of miR-122 and high expression of aldolase A [141]. Therefore, the DIO3OS/miR-122/aldolase A axis could be exploited as a therapeutic target in PDAC progression.

Glyceraldehyde 3-phosphate dehydrogenase (GAPDH), another vital enzyme in glycolysis, exhibits increased expression in PDAC at both the mRNA and protein levels. Acetylation of lysine 254 augments the activity of GAPDH in glycolysis and promotes the proliferation of cancer cells [35]. Increased GAPDH mRNA and protein expression is associated with increased glycolytic flux in PDAC. It was demonstrated that GAPDH exerts its effects through DNA repair, autophagy, apoptosis, iron metabolism and transcriptional regulation in addition to catalysis in glycolysis, so this enzyme can be detected in the cytosol, membrane, nucleus, Golgi, and endoplasmic reticulum. Moreover, mutant p53 enhances glycolytic GAPDH activity and induces the formation of the SIRT1-GAPDH complex, which can stabilize cytosolic GAPDH for glycolysis and induce tumor growth and survival in PDAC [10]. The natural product koningic acid (KA) is an established selective inhibitor of GAPDH that exerts bioactivity mainly on tumors, with little effect on normal tissue. It inhibits the activity of GAPDH, leading to low glycolytic flux and a low cytotoxic response in highly glycolytic tumor cells [142]. Another inhibitor of GAPDH is iodoacetate; when PANC-1 cells are cultured with iodoacetate, low glycolytic flux is observed, accompanied by reduced cell survival. However, there is little alteration in the signaling machinery [143].

Phosphoglycerate kinase (PGK), as the first enzyme in glycolysis that produces ATP, includes two forms of PGK, the extensively expressed form PGK-1 and the testis-expressed form PGK-2. It has been demonstrated by immunohistochemistry that PGK-1 is highly expressed in PDAC [144]. Interestingly, PGK-1 has been proven to affect DNA duplication and repair in the mammalian nucleus. The mRNA and protein expression of PGK-1 is relevant to poor outcomes and a poor prognosis [145]. Accordingly, PTMs play vital roles in regulating the function of PGK-1 during tumorigenesis. More specifically, promoter methylation has been negatively associated with the mRNA expression of PGK-1; phosphorylation of the PGK-1 protein was associated with the clinical outcomes of PDAC patients [144]. Interestingly, another study reported that pyruvate dehydrogenase kinase 1 (PDHK-1) is phosphorylated by mitochondria-translocated PGK-1 (acting as a protein kinase), which activates PDHK-1 and augments glycolysis but inhibits OXPHOS [146]. Under glutamine deprivation and hypoxia in cancers, PGK-1 has an enhanced interaction with the acetyl-transferase ARD1, leading to acetylation of PGK-1 at lysine388. Subsequently, PGK-1 (acting as a protein kinase) phosphorylates Beclin1 without affecting the formation of Beclin1/VPS34/ATG14L, which causes an altered conformation and increased activity of VPS34 as well as augmented autophagy for tumor homeostasis [147]. However, HIF-1α upregulates the expression of PGK-1 but not its subcellular distribution under hypoxia. Increased expression of PGK-1 can result in the catalytic phosphorylation of troxacitabine, converting troxacitabine into the triphosphate form and causing increasing cytotoxicity to PANC-1 cells [148]. When PDAC is treated with troxacitabine, creating PGK-1 overexpression conditions to increase the cytotoxicity of troxacitabine may be a potent method for PDAC therapy. In a study, when FOXM1 expression was knocked down, PGK-1 levels were also decreased significantly, similar to the changes in LDHA levels mentioned before; however, the author did not specify the concrete mechanisms related to FOXM1 and PGK-1 [39]. Nuclear Factor of Activated T Cells 5 is highly expressed in PDAC patients and is associated with tumor progression by positively modulating PGK-1 [149]. *SMAD4* and *PTEN* are distinctly silenced in PDAC. Loss of *SMAD4* in PDAC is responsible for the high glycolytic capacity and tumor progression by upregulating PGK-1 expression, which has been shown to have dual roles in glycolytic catalysis and transcription factor activity in metastasis. Nuclear PGK-1 induces EMT by repressing E-cadherin expression, thus contributing to the migratory and metastatic potential. Cytoplasmic PGK-1 affects the metabolic type of PDAC by regulating the ratio of glycolysis and mitochondrial oxidative phosphorylation. Moreover, *SMAD4*-silenced PDAC patients can be classified by the subcellular localization of PGK-1 into nuclear PGK-1 positive or negative and high or low cytoplasmic PGK-1 to differentiate patient prognoses [150]. The new classification and interaction between *SMAD4* and PGK-1 suggests some predictions and an instructive strategy for PDAC treatment, but these conclusions need to be thoroughly confirmed before a clinical application is proposed. In addition, PGK-1 is activated through autophosphorylation at tyrosine 324 (Y324), which enhances glycolysis and proliferation. PTEN exerts its protein phosphatase effect and dephosphorylates PGK-1, but the loss of PTEN found in glioblastoma is strongly associated with a poor prognosis [151]. Thus, regulating the PTMs of PGK-1 to block glycolysis and proliferation in tumors may provide novel methods for cancer therapy.

Phosphoglycerate mutase (PGAM) displays relatively high expression and activities in many malignant cancers, including PDAC. Via proteomic techniques, PGAM-1 is statistically indicated to be associated with a relatively poor prognosis in PDAC [152]. After knockdown of PGAM-1 expression, decreased glycolytic flux and reduced cell invasion were observed by detecting 3-PG and 2-PG levels and performing invasion experiments, respectively, but the association with proliferation was independent [36]. A study reported that PGAM-1, which is downstream of the PI3K/Akt/mTOR pathway, promoted EMT by stimulating the Wnt/β-catenin pathway, offering a potential application in PDAC through targeting a related signaling pathway to regulate the activity or expression of PGAM [153]. Moreover, acetylated PGAM-1 displays augmented enzymatic activity, and SIRT-1, an NAD^+^-dependent deacetylase, inhibits enzymatic activity by deacetylating PGAM [154]. Another NAD^+^-dependent deacetylase, SIRT-2, induces deacetylation at lysines 100/106/113/138 of PGAM-2 to inhibit enzymatic activity and repress tumor proliferation [155]. Interestingly, the phosphorylation of PGAM-1 at tyrosine 26 (Y26) was proven to activate enzymatic activity to alter glycolytic flux and induce migration [156]. Therefore, targeting PTMs, such as dephosphorylation and deacetylation, at some sites in PGAM-1 could attenuate PGAM-1 enzymatic activity and tumorigenesis for PDAC treatment. Recently, a newly developed and promising compound, KH3, was found to act as an allosteric suppressor of PGAM-1, showing satisfactory drug effects to downregulate glycolysis and cell proliferation with limited cytotoxicity to PDAC cells [152].

Enolase is the enzyme that generates PEP in glycolysis, and there are five forms of enolase in mammalian tissue composed of three immunological subunits, alpha, beta, and gamma. The alpha subunit exists in many tissues; the beta subunit is present only in skeletal and heart muscles, and the gamma subunit is localized in neurons [157]. Alpha enolase (ENO-1) levels are elevated in PDAC cells [37, 157, 158], which correlates with the migration and invasion of PDAC cells by inducing plasminogen activation into plasmin to degrade the compact ECM in a plasminogen-dependent process, and this process can be strongly blocked by using an adeno-associated virus/anti-ENO-1 antibody construct [159]. In addition, aberrant expression of ENO-1 is positively correlated with *Ki67* and negatively correlated with *p53* in PDAC, which indicates that ENO-1 exerts its effects on proliferation and metastasis by regulating the *Ki67* and *p53* pathways. In addition, increased ENO-1 expression is relevant to HIF-1α under hypoxia [37]. ENO-1 acts as the receptor of plasminogen in addition to a glycolytic enzyme, and silencing ENO-1 attenuates adhesion, invasion, and metastasis in PDAC [160]. Similarly, PTMs can also regulate ENO-1 activity, and phosphorylating ENO-1 peptide-MHC complexes at serine 419 can induce T cell signaling and autoantibody production in PDAC [158]. Accordingly, SF2312 produced by Micromonospora actinomycetes was first used as an antibiotic and is considered a specific inhibitor of enolase [161]. ENO-1 DNA vaccination has been established for PDAC, and additional treatments, such as ENO-1 inhibitor application, immune cell activation, or chemotherapy, in combination with ENO-1 vaccination could amplify the therapeutic response in PDAC [162].

### Metabolic reprogramming interactions

The oxidative phenotype exists in PDAC, and cells with the glycolytic phenotype can also choose OXPHOS to sustain metabolic needs when glycolysis is inhibited in PDAC, as we mentioned before. Increased OXPHOS can be characterized by activated enzymes and increasing levels of products of the TCA cycle; moreover, mitochondrial dynamics and the mitochondrial membrane potential can be evaluated to confirm this phenotype [100]. The ROS level is the major factor that affects the tumorigenesis of PDAC, and low levels of ROS, which are produced by respiratory complex IV, activate vital redox signaling pathways; additionally, respiratory complexes I, II, and III produce superoxide, which may cause oxidative stress and mitochondrial dysfunction [163]. Mitochondrial respiration unifies several important bioenergetic pathways; it produces precursors for lipid, amino acid, and nucleotide biosynthesis and contributes to glutamine metabolism (Fig. 2). In addition to glucose, glutamine, a dispensable amino acid, is another nutrient fuel for cancer energy production and biosynthesis. Of note, glutamine deprivation is greatly associated with tumor suppression [164]. Glutamine is transferred into cells through alanine/serine/cysteine-preferring transporter 2 (ASCT2) in PDAC cells [165], and then it is converted into glutamate by glutaminase (correlated with EMT in hepatocellular carcinoma and encoded by *GLS1* [166]) and metabolized to α-ketoglutarate as a carbon donor for the TCA cycle via amino acid transaminase or glutamate dehydrogenase, which is also accepted as anaplerosis. In addition, glutamine provides nitrogen for nucleotide synthesis and synthesis of other dispensable amino acids (such as arginine, asparagine, serine, alanine, aspartate, glycine, and cysteine) [167]. De novo lipogenesis is a prominent feature of PDAC cells and is essential for fatty acid biosynthesis. Lipid metabolism provides aggressive PDAC cells with enough lipids for the cell membrane, energy production, and second messenger and signaling molecule generation. Another intermediate in the TCA cycle, citrate, is transformed into acetyl-coenzyme A (CoA) via ATP-citrate lyase (ACLY) and then converted into malonyl-CoA, which is mediated by acetyl-CoA carboxylase (ACC) (encoded by *ACACA* or *ACACB*), finally producing fatty acids through fatty acid synthesis (FASN). The expression of ACLY, ACC, and FASN is augmented in some PDAC patients, who show a shortened survival time, chemoresistance, and a poor prognosis. Downregulating the activity and expression of these proteins reduces lipid generation while suppressing tumor proliferation and inhibiting tumorigenesis [168–170]. In hepatocellular carcinoma, it has been proven that inhibiting the AMPK-induced phosphorylation of ACC causes larger lesions and progression in hepatocellular carcinoma. The opposite results are observed when treatment with the ACC inhibitor ND-654, which imitates the effects of phosphorylating ACC, is applied [171]. Expression of *FASN* is mediated by transcriptional factor sterol regulatory element-binding protein 1c (SREBP1c), and downregulating PI3K and MAPK pathways can inhibit SREBP1c to decrease *FASN* transcription and suppress lipogenesis and PDAC progression. Furthermore, after FASN and SREBP1c expression was analyzed, SREBP1c was demonstrated to directly or coordinately regulate enzymes and FASN in lipogenesis [172]. The hexosamine biosynthetic pathway (HBP) is greatly interlinked with other metabolic pathways, such as glucose, lipid, nucleotide, and amino acid metabolism, in PDAC. The end product of the HBP, uridine diphosphate N-acetyl glucosamine (UDP-GlcNAc), is generated from 3 materials, glucose, glutamine, and glucosamine, and acts as a substrate for O-GlcNAcylation. The HBP and glycolysis share the first two steps from glucose to F-6-P generation, and then F-6-P and glutamine are converted into glucosamine-6-phosphate via glutamine fructose-6-phosphate amidotransferase (GFAT); meanwhile, glucosamine is converted into glucosamine-6-phosphate by GlcNAc kinase. Then, glucosamine-6-phosphate is catalyzed to UDP-GlcNAc with the assistance of intermediates including acetyl-CoA and UTP from de novo lipogenesis and nucleotide metabolism, respectively [173]. O-GlcNAcylation is a PTM on some proteins that leads to metastatic potential and aggressive reactions, and transcriptional regulation is widely disrupted (both O-GlcNAcase and O-GlcNAc transferase levels are increased) in PDAC to promote tumor growth [174]. The activated HBP is not only highly interconnected with other metabolic pathways but is also closely related to growth, PTMs, invasion, EMT, and aggressiveness in PDAC [173].

## Clinical applications of glycolysis in pancreatic cancer

### Detection

Recent detection approaches for PDAC include biomarker evaluations, imaging assays, and oncogene mutation evaluations, but none of these can act as an independent confirmatory diagnostic measurement. The anatomically inaccessible location of the pancreas leads to an incomplete diagnosis [29]. Some serum biomarkers, such as CA-125/CEA/CA19–9, are correlated with poor surgical outcomes and a poor prognosis [175–177]. However, conventional tumor markers, such as CA19–9 and CEA, lack sensitivity and specificity. However, traditional imaging examinations such as CT and magnetic resonance imaging (MRI) can only reflect the visual tumor size measured by a computer [178]. These measurements lack the foresight needed to predict the occurrence of PDAC and provide additional information related to malignancy. Thus, analysis of metabolic rearrangements in glycolysis could be considered a relatively precise diagnostic means for the detection of the metabolic phenotypes of pancreatic cancer to indicate malignancy. Specifically, this section summarizes alterations in enzymes and serum metabolites in glycolysis differing between PDAC patients and healthy people to explore if these parameters could be prospective measurements for PDAC (Table 1).

**Table 1:** Enzymes and metabolites in glycolysis for the detection of PDAC

Molecules	Sources	Levels	Refs	Detection methods
**Enzymes**				
HK-2	Gene profiles of PDAC patients (*n* = 143)	up	[179]	Western blot [180], immunochemical staining [181], MS [182]
PFK-p	GeneChip hybridization of paired normal and tumor specimens from PDAC patients (*n* = 36)	up	[183]	Immunohistochemistry [184], capillary electrophoresis [185]
PKM-2	Tissue microarray of PDAC patients (*n* = 90) and the Oncomine database	up	[186]	LC-MS/MS [187], western blot [188], immunochemical analysis [189]
LDH-A	Gene profiles of PDAC patients (n = 143)	up	[179]	Immunohistochemistry [188], serum quantification [190]
Aldolase	Tissue microarray containing paired cancer and normal tissue specimens from PDAC patients (*n* = 96)	up	[137]	Immunohistochemistry [191], western blot [137]
GAPDH	2-D gel electrophoresis of paired cancer and normal tissue specimens from PDAC patients (*n* = 10)	up	[192]	Immunoblotting [142], western blot [193], northern blot [194]
PGK-1	2-D gel electrophoresis of paired cancer and normal tissue specimens from PDAC patients (*n* = 63)	up	[145]	Protein microarray of serum [195], western blot [148]
PGAM	Immunohistochemistry of paired cancer and normal tissue specimens from PDAC patients (*n* = 54)	up	[153]	Western blot [37], 2-D immunoblotting and LC-MS/MS [196]
ENO-1	2-D gel electrophoresis of paired cancer and normal tissue specimens from PDAC patients (n = 10)	up	[192]	Immunohistochemistry [37], western blot [197]
PGI	Microarray analysis of a human PDAC xenograft in a rat model	up	[198]	Western blot and immunoprecipitation [199]
TPI	Gene profiles of PDAC patients (n = 143)	up	[179]	–
**Metabolites**				
Glucose	Glycemic profiles of PDAC patients (*n* = 219)	up	[200]	Blood draw for fasting blood glucose
Lactate	Pancreatic juice from PDAC patients (*n* = 79) and non-PDAC patients (*n* = 27)	up	[201]	ERCP for pancreatic juice analysis
**Others**				
GLUTs	Gene profiles of PDAC patients (n = 143)	up	[179]	Immunohistochemistry

TPI catalyzes the reaction of glycolysis in a non-KRAS-dependent manner and shows no correlation with PDAC progression

Augmented enzyme expression and activity indicate an increase in glycolysis, which also acts as a vital sign of PDAC [202]. These changes in proteins can be detected by mass spectrometry (MS), which identifies molecules by measuring the mass-to-charge ratio, and the instrument includes an ion source, a mass analyzer, a detector, and a data system [203]. Moreover, GLUTs accumulate in PDAC, so more glucose is conveyed into the cytoplasm through these transporters. Upregulation of GLUT expression is generally associated with a poor prognosis and can be detected by the MS technique [26, 204]. Similarly, this tool has been developed to analyze proteomics, including large-scale protein expression and PTMs in glucose metabolism. Dysfunction of the enzymes in glycolysis is usually induced by PTMs, which are involved in the formation, proliferation, and invasion of PDAC, such as acetylation, phosphorylation, O-methylation, and glycosylation, and these modifications can also be detected by LC-MS/MS. [205] However, not all molecules can be detected by MS, and biomarkers in glycolysis with a low abundance in PDAC, such as HIF-1α, cannot be detected by MS. [26].

Presently, many researchers have deemed metabolism to be a vital characteristic for detecting and treating pancreatic cancer, so the metabolic tumor burden (MTB), especially total lesion glycolysis (TLG), is a new parameter to discriminate pancreatic carcinoma from benign pancreatic diseases. In addition to discriminating PDAC, TLG can also predict clinical outcomes and prognosis because it is related to poor OS and recurrence-free survival (RFS) in PDAC [206]. Interestingly, these indexes, such as the MTB and metabolic tumor volume (MTV), are detectable by 18-fluoro-deoxyglucose positron emission tomography computed tomography (^18^FDG-PET/CT) [20], which uses ^18^FDG as a tracer because of the elevated uptake of glucose. Notably, ^13^C-labeled metabolites can translate into cancer cells for reprogramming metabolism, such as glycolysis, and the metabolic process can be detected by hyperpolarized magnetic resonance spectroscopy (MRS), which can be used to discover multiple metabolic processes via labeled metabolites of glucose, amino acids, lipids, and nucleotides in PDAC [29]. For example, 1-^13^C-labeled pyruvate was converted into lactate in a preclinical PDAC model and detected by hyperpolarized MRS to discriminate cancer cells from normal cells [207]. The safety of hyperpolarized MRS has been proven in clinical trials for prostate cancer [208]. In addition, a new study reported that the specific preoperative neutrophil-lymphocyte ratio in the serum was associated with OS after resection in PDAC patients [209].

.In summary, conventional imaging examinations such as CT and MRI can be used for surgical resectability and range; biomarkers in body fluids, such as the serum and urine, can be evaluated to monitor for PDAC; the expression of glycolytic enzymes and regulators and the concentrations of glycolytic metabolites in the serum can be used to predict prognosis and malignancy; PET and MRS can be used to evaluate the metabolic properties and metastasis of pancreatic cancer; and TLG and MTV can be used to predict prognosis and clinical outcomes. We believe that an integrated method together with genomic profiling of PDAC may provide a prognostic and personalized diagnosis for PDAC patients.

### Therapy

Traditional treatment strategies include chemotherapy, radiation therapy, and molecular targeted therapy. However, surgery is the only possibly curative treatment [210]. After surgery, neoadjuvant chemoradiation is increasingly used to treat PDAC patients [211]. Furthermore, metabolic rearrangements can not only reflect characteristics and hallmarks for diagnosis and prognosis but also lead to the development of new therapeutic strategies as biomarkers for PDAC. This review summarizes inhibitors of glycolytic enzymes and regulators to explore whether they could be prospective treatments for PDAC (Table 2).

**Table 2:** Enzymes in glycolysis for possible PDAC therapy

Enzymes	Compounds or methods	Introductions	Ref
HK	2-DG	Inhibitor of HK, acts as a glucose analog phosphorylated by HK that then blocks glycolysis	[117]
	3-BP	Inhibitor of HK, suppresses activity of HK and glycolysis	[118]
	Novel MJ analog	Inhibitor of HK, disrupts VDAC and HK-2 interactions on the mitochondrial membrane and then inhibits glycolysis	[119]
	Downregulate HOTAIR	Inhibits the expression of HK in both the serum and tumor tissue to suppress glycolysis and tumor growth	[120]
PFK	3-PO	Inhibits PFKFB3, reduces the synthesis of F-2,6-BP and then inhibits PFK activity	[125]
	Activates AMPK	Attenuates phosphofructo-2-kinase activity and increases fructose-2-biphosphatase activity to inhibit PFK	[123]
	Target miR-135	Inhibits PFK, can construct a glutamine-deficient condition in combination with a glutaminase inhibitor	[127]
PK	Downregulate PKM-2	Resumes p38 mitogen–activated protein kinase activity and enhances gemcitabine-resistant cell apoptosis	[130]
	TEPP-46	Activates the tetramer of PKM-2 and impairs tumor growth and proliferation	[129]
	TEPP-46 and FX-11	Significant antitumor effect and limited toxicity in finished phase III trial	[129]
LDH	FX-11	Inhibitor of LDH-A, suppresses glycolysis and tumor growth	[133]
	Target FOXM1	FOXM1/LDH-A pathway promotes glycolysis and lactate production	[39]
	Novel NHI inhibitors	Targets LDH-A, inhibits glycolysis, growth, and invasion in PDAC	[135]
	TEPP-46 and FX-11	Significant antitumor effect and limited toxicity in finished phase III trial	[129]
Aldolase	TX-2098	Suppresses HIF-1α, GLUT1, and Aldolase A, leads to distinct antitumor effect on PDAC xenograft model	[139]
	NDB	Inhibitor of Aldolase A, has high affinity and resistance to hydrolysis, suppresses glycolysis and tumor growth	[140]
GAPDH	KA	Inhibitor of GAPDH, leads to low glycolytic flux in cancer cells and little cytotoxic to normal tissue	[142]
	Iodoacetate	Inhibitor of GAPDH, leads to reduced glycolysis and cell survival	[143]
PGK	Target SMAD4	Inhibits expression and subcellular localization of PGK-1 to decrease EMT and glycolysis in PDAC	[150]
	Activate PTEN	Dephosphorylates PGK-1 at Y324 to block glycolysis and proliferation in cancer cells	[151]
PGAM	KH3	Allosteric suppressor of PGAM-1, represses glycolysis and cell proliferation with limited cytotoxicity	[152]
	Target PTMs	Targets PTMs such as PGAM-1 dephosphorylation and deacetylation to attenuate enzymatic activity	[154] [155] [156]
Enolase	Sodium fluoride	Inhibitor of enolase, decreases glycolysis and invasion in PDAC	[212]
	SF2312	Specific inhibitor of enolase, reduces glycolysis and invasion	[161]
PGI	6-phosphogluconic acid	Possible inhibitor of PGI, induces low PGI activity and attenuates glycolysis	[212]
**Others**			
*KRAS*	AMG-510	First inhibitor of KRAS, causes a decline in KRASG12C tumor growth	[213]
*BRCA1/2*	Olaparib	Approved by the FDA for BRCA1/2-mutant metastatic PDAC	[214]

Recently, the fructo-1,6-bisphosphatase (FBP)-mediated rate-limiting step in gluconeogenesis has been reported to inhibit the Warburg effect and *KRAS* signaling. Furthermore, FBP-2 and FBP-1 were found to attenuate soft tissue sarcomas (STS) and breast cancer, respectively. In STS, the authors re-expressed FBP-2 through overexpression vectors and found that FBP-2 inhibited glycolysis in the cytoplasm and suppressed mitochondrial respiration, biogenesis and the tricarboxylic acid (TCA) cycle in the nucleus by inhibiting the function of the transcription factor c-Myc in the nucleus. In breast cancer, restoration of FBP-1 expression intensely inhibited glycolysis by catalyzing the function and silencing activity of HIF [34]. However, the exact role of FBP-2 in PDAC is still obscure; the precise mechanism remains unknown. In addition, targeted therapy aimed at common gene mutations consistently exhibits a lack of efficacy because the most commonly mutated genes in pancreatic cancer are known to be *KRAS* and *TP53,* which can hardly be made into drug targets [3]. Promisingly, AMG-510, the first inhibitor of *KRAS* in clinical development, caused a decline in *KRAS*^G12C^ tumor growth and augmented the efficacy of chemotherapy and targeted agents [213]. Moreover, in clinical data, approximately 8% of PDAC samples had germline and somatic mutations in the DNA damage repair genes *BRCA2*, *PALB2*, and *ATM* [6]. Inspiringly, a recent study noted that olaparib, a poly ADP-ribose polymerase (PARP) inhibitor, prolonged PFS among germline *BRCA*-mutant PDAC patients [214–216]. Olaparib has been approved by the FDA for metastatic pancreatic cancer, breast cancer and recurrent ovarian cancer with *BRCA*1/2 mutation [214]. Although olaparib exhibits a therapeutic effect, the application of olaparib is limited to patients with mutations in the DNA damage repair gene *BRCA*2, and further studies will be required to identify a detailed approach for treating PDAC.

## Conclusion

In conclusion, we first discussed the cause and characteristics of glycolysis in PDAC, especially the microenvironment and metabolic subgroup. Next, we summarized the vital kinases in the glycolytic process. Notably, glycolysis in PDAC can be easily suppressed by inhibiting the activation of critical enzymes such as HK, PFK, and PK. These kinases catalyze the rate-limiting steps in glycolysis, so we analyzed diagnostic and therapeutic means focused on these enzymes to target PDAC. Moreover, we also presented the latest findings related to clinical therapy and the diagnosis of PDAC. However, the precise molecular mechanisms of glycolysis and PDAC are still unknown. Therefore, the processes of clinical applications and molecular mechanisms should also be explored to maximize the proportion of PDAC patients who will derive benefit.

## Data Availability

Data available upon request.

## Abbreviations

1,3-DPG: 1,3-diphosphoglyceric acid
2-DG: 2-deoxy-D-glucose
3-BP: 3-bromopyruvate
3-PG: 3-phosphoglycerate
3-PO: 3-(3-pyridinyl)-1-(4-pyridinyl)-2-propen-1-one
ACLY: ATP-citrate lyase
AMPK: cAMP-dependent protein kinase
aPSCs: Activated pancreatic satellite cells
ASCT2: Alanine/serine/cysteine-preferring transporter 2
ATP: Adenosine triphosphate
CAF: Cancer-associated fibroblast
CoA: Acetyl-coenzyme A
CT: Computed tomography
DHAP: Dihydroxy-acetone phosphate
ECM: Extracellular matrix
F-1,6-BP: Fructose-1,6-biphosphate
F-2,6-BP: Fructose-2,6-biphosphate
F-6-P: Fructose-6-phosphate
FASN: Fatty acid synthesis
FBP: Fructo-1,6-bisphosphatase
FOXM1: Forkhead box protein M1
FX-11: 3-dihydroxy-6-methyl-7-(phenylmethyl)-4-propylnaphthalene-1-carboxylic acid
G-6-P: Glucose-6-phosphate
GA3P: 3-phosphoglyceraldehyde
GAPDH: Glyceraldehyde 3-phosphate dehydrogenase
GFAT: Glutamine fructose-6-phosphate amidotransferase
GK: Glucokinase
GLUT: Glucose transporter
HBP: Hexosamine biosynthetic pathway
HK: Hexokinase
HMGCR: 3-hydroxy-3-methylglutaryl coenzyme A reductase
HOTAIR: Hox transcript antisense RNA
KLF4: Krüppel-like factor 4
LDH: Lactate dehydrogenase
MCT: Mono-carboxylate transporter
MMP-2: Metalloproteinase-2
MRI: Magnetic resonance imaging
MRS: Magnetic resonance spectroscopy
MS: Mass spectrometry
MTB: Metabolic tumor burden
MTV: Metabolic tumor volume
OS: Overall survival
OXPHOS: Oxidative phosphorylation
PARP: Poly ADP-ribose polymerase
PDAC: Pancreatic ductal adenocarcinoma
PDHK: Pyruvate dehydrogenase kinase
PEP: Phosphoenolpyruvate
PET: Positron emission tomography
PFK: Phosphofructokinase
PFKFB: Phosphofructo-2-kinase/fructose-2,6-biphosphatase
PGAM: Phosphoglycerate mutase
PGI: Phosphohexose isomerase
PK: Pyruvate kinase
PSC: Pancreatic stellate cell
PTM: Posttranslational modification
qPSCs: Quiescent pancreatic stellate cells
RAGE: Receptor for advanced glycation end products
RFS: Recurrence-free survival
ROS: Reactive oxygen species
SREBF2: Sterol regulatory element binding transcription factor 2
SREBP: Sterol regulatory element-binding protein
STS: Soft tissue sarcomas
TCA: Tricarboxylic acid
TEPP-46: Thieno [3,2-b] pyrrole [3,2-d]pyridazinone
TIGAR: TP53-induced glycolysis and apoptosis regulator
TLG: Total lesion glycolysis
TME: Tumor microenvironment
TPI: Triose phosphate isomerase
UDP-GlcNAc: Uridine diphosphate N-acetyl glucosamine
VDAC: Voltage-dependent anion channel
